# Numerical modelling of ZnO-based solid-state dye-sensitized solar cells employing CdTe and kesterite Cu_2_XSnS_4_ (X = Fe, Ba and Mn) hole transport layers

**DOI:** 10.1098/rsos.251606

**Published:** 2026-11-11

**Authors:** Minhajul Abedin, Imtiaz Ahamed, Md. Zahid Hasan, M. S. H. Choudhury

**Affiliations:** Department of Electrical and Electronic Engineering, International Islamic University Chittagong, Chattogram, 4318, Bangladesh

**Keywords:** solid-state, hole transport layer, solid-state dye-sensitized solar cells, kesterite, quantum efficiency, current density, SCAPS-1D

## Abstract

This study investigates the performance of solid-state dye-sensitized solar cells (ssDSSCs) employing four hole transport layer (HTL) candidates: cadmium telluride (CdTe) and three kesterite materials—copper-barium-tin sulfide (CBTS), copper-iron-tin sulfide (CFTS) and copper-manganese-tin sulfide (CMTS). Electrical and optical parameters collected from the literature were incorporated into SCAPS-1D for device modelling. A comprehensive analysis examined the effects of HTL thickness, temperature, absorber and electron transport layer (ETL) thickness, capacitance, parasitic resistance and doping concentration on device performance. The results demonstrate that CdTe is a highly promising HTL, with the optimized fluorine-doped tin oxide (FTO)/ZnO/ruthenium complex dye (N719)/CdTe/Pt heterostructure achieving a power conversion efficiency (PCE) of 21.58%, a fill factor (FF) of 87.86%, an open-circuit voltage (*V*_OC_) of 1.106 V and a current density (*J*_SC_) of 22.21 mA cm^−^², exceeding previously reported values. Among kesterite-based HTLs, CBTS and CFTS exhibited notable PCEs of 16.42 and 14.80%, respectively, outperforming comparable studies. These findings indicate that CdTe, CBTS and CFTS are strong candidates for ssDSSC applications, offering enhanced efficiency and stability. Notably, CBTS emerges as a low-cost, Earth-abundant alternative with favourable band alignment and high thermal stability. This work provides valuable insights for advancing efficient and sustainable ssDSSC technologies.

## 1. Introduction

In recent years, the pursuit of sustainable energy alternatives has gained significant momentum owing to growing global concerns regarding climate change, energy security and the limited availability of conventional fossil fuel resources. To tackle these pressing issues, renewable energy technologies have gained significant interest over the past century. Renewable technologies, such as hydrogen energy, photovoltaics (PVs), bioenergy and wind power, have progressed substantially, but their widespread deployment remains limited owing to intermittency, high capital costs, storage limitations and challenges related to grid integration and sustainable material availability [1,2]. To address these constraints, current research focuses on integrated energy systems that combine renewable generation with chemical fuels and advanced storage solutions to enhance reliability and operational flexibility [3]. Material innovation plays a crucial role in this transition, as improvements in catalysts, photoactive layers and storage media directly affect system efficiency and scalability [4–6].

Among contemporary renewable energy technologies, solar cells (SCs) have emerged as one of the most significant and widely investigated renewable energy solutions. It is a promising and consistent alternative to meet high energy demand. It also enables the generation of clean, renewable energy while causing minimal harm to the environment, making it a cornerstone in the transition towards sustainable energy systems. The core component of a SC is a p–n junction, which is essentially composed of semiconductive elements. Solar cells are categorized as organic, inorganic, homojunction, heterojunction and so on based on the semiconductive material employed and the number of junctions between the semiconductors. Therefore, semiconductors are the fundamental component of PV systems because of their special electronic characteristics, which allow sunlight to be converted into electrical energy [7]. Semiconductors are distinguished by their well-structured energy bandgap and intermediate electrical conductivity, which enable the effective absorption and manipulation of photons to produce electron–hole pairs. The design and performance of SCs are critically dependent on the properties of the semiconducting materials used [8,9]. Solar cell technology initially based on crystalline silicon with a doped homojunction structure, known for its efficiency and robust performance, dominated the market.

In recent years, dye-sensitized SCs (DSSCs), classified as third-generation PV devices, have emerged as promising alternatives to conventional silicon-based SCs [10,11]. Introduced through the pioneering work of O'Regan and Grätzel in 1991, DSSCs offer an efficient and affordable way to convert solar light into electrical power [12]. DSSCs are commonly based on semiconducting TiO_2_ and ZnO materials. Nanocrystalline titanium oxide (TiO_2_) and zinc oxide (ZnO) are widely used semiconductor materials in DSSCs owing to their excellent electron transport properties, optical transparency and low cost [13–16]. One key component of DSSCs is the sensitizer dye, which plays a crucial role in light absorption and electron injection. During the selection of dyes, several critical criteria must be considered to optimize efficiency and stability. These include the requirement for the dye to exhibit luminescence and have absorption spectra covering UV–visible (UV-vis) and near-infrared (NIR) regions. Additionally, the highest occupied molecular orbital (HOMO) should be distant from the TiO_2_ or ZnO conduction band, while the lowest unoccupied molecular orbital (LUMO) should be close and higher in energy. The HOMO should also be lower than that of redox electrolytes [17]. TiO_2_ and ZnO have been sensitized with different organic sensitizers such as eosin dye and rose Bengal dye. Ruthenium complex dye (N719) gave the best performance as a sensitizer. However, the energy conversion efficiency of the DSSCs is still only approximately 10–14% [18,19].

In DSSCs, the fabrication process involves several critical stages. A transparent conductive oxide (TCO) substrate, typically fluorine-doped tin oxide (FTO), is used as the working electrode. It is then coated with a layer of semiconducting material, often titanium dioxide (TiO_2_) or zinc oxide (ZnO) nanoparticles, using different techniques such as doctor blading, spin coating, spray coating, sol–gel, electrophoretic deposition and chemical vapour deposition [20,21]. This layer serves as the photoanode and is essential for efficient electron transfer [22]. Sensitization follows, with organic or inorganic dyes absorbing light and injecting electrons into the conduction band, initiating electron transport. A redox electrolyte, typically containing iodide/triiodide ions, regenerates the dye and facilitates electron transport to the counter electrode. The counter electrode, usually coated with a catalyst layer like platinum, reduces triiodide ions to iodide ions. To optimize the properties of deposited materials and thin films, various post-deposition methods, such as annealing, compression and hot-compression, are used [23,24]. These steps collectively enable the conversion of sunlight into electricity in DSSCs. DSSCs are appealing owing to their several advantages, including low production costs, ease of fabrication, lightweight design and increased efficiency in low-light conditions [25]. Additionally, their transparency, colour versatility and mechanical flexibility enable a wide range of potential applications in optoelectronic devices. Unlike amorphous silicon SCs, which tend to degrade under prolonged sunlight exposure, DSSCs exhibit greater photostability [19,26,27]. However, despite their promising features, they come with major drawbacks that hinder their overall efficiency, including a narrow light absorption spectrum, issues related to charge recombination and corrosion of the counter electrode. The use of liquid electrolytes, which are prone to leakage and evaporation, can also significantly impair cell performance and long-term stability. To address this problem, solid-state materials can be used instead of liquid electrolytes [28,29].

Solid-state DSSCs (ssDSSCs) are a type of DSSC in which a solid-state hole transport material is used in place of the liquid electrolyte. This substitution significantly enhances the thermal and chemical stability of the device, thereby improving its durability and making it more suitable for practical applications under demanding environmental conditions [30,31]. In pursuit of enhanced performance, researchers have explored a wide range of hole transport materials (HTMs), including organic and inorganic compounds, ionic conductors, perovskites and doped molecular systems. The incorporation of these novel HTMs not only contributes to higher efficiency but also supports flexible and sustainable device architectures. Current literature reviews underscore the importance of HTMs in driving both efficiency and longevity in ssDSSCs, marking them as key components in the development of reliable and scalable solar technologies [32–34]. Recent studies have shown notable advancements in ssDSSC technology, with efficiency levels exceeding 10% and approaching the 15% mark in some instances [35]. The ssDSSC with Cu(II/I) complex as the HTM attained an 11% conversion efficiency under air mass 1.5 global (AM1.5G) conditions [36]. In contrast, the conversion efficiencies using Cs_2_SnI_6_, PEDOT and spiro-MeOTAD as HTM were 7.1, 7.7 and 7.8%, respectively [37–39].

Nowadays, researchers are increasingly turning to simulation-based research in the development of high-efficiency SCs, primarily owing to the time-consuming and intricate nature of their fabrication processes. Traditional fabrication processes of SCs are often complex, time-intensive and financially burdensome, particularly when procedural errors occur. In contrast, simulators offer a faster, more cost-effective and safer alternative for researchers [40]. Through virtual testing and design optimization, simulation tools eliminate the need for expensive materials, intricate production set-ups and specialized laboratory environments, thereby significantly accelerating the research and development timeline compared with hardware-based research. In addition, first-principles methods, such as density functional theory, enable precise prediction of electronic, optical, thermal and structural properties of PV materials, providing essential insights for material selection and device optimization [41–45]. SCAPS-1D, wxAMPS, COMSOL and other simulation software are among the most widely used tools for designing and analysing SCs [46]. Using the SCAPS-1D simulator, a maximum PCE of 20.73% was theoretically reported for the ITO/PCBM/N719 dye/PEDOT:PSS/Pt configuration and 28.22% for the WS_2_/N719/copper-barium-tin sulfide (CBTS)/Au configuration [47,48]. Many other researchers have also investigated various ssDSSC configurations, with most reported efficiencies ranging between 5 and 15% [49–53].

A class of materials comprising sulfide minerals, specifically known as kesterite and stannite compounds, has gained significant attention as SC absorbers. These materials are recognized for their low cost, abundance in the Earth's crust and high stability, positioning them as promising candidates for PV applications [54,55]. Depending on their particular composition, kesterite materials (Cu_2_XSnS_4_) usually have an average bandgap of 1.0–1.5 eV. These bandgaps enable effective absorption of the solar spectrum and align with the Shockley–Queisser limit's ideal bandgap for SCs, establishing kesterites as a compelling option for thin-film SCs [56,57]. In relation to their use as a hole transport layer (HTL), an experimental study demonstrated the application of kesterite, specifically copper-zinc-tin sulfide (CZTS), as an HTL in perovskite SCs [58]. Additionally, other groups demonstrated the use of various other kesterite materials as HTL in perovskite SCs using the wxAMPS and SCAPS-1D simulators. To the best of our knowledge, there have been no efforts to employ kesterite as a solid-state hole transport material in DSSCs, with the exception of a study conducted by Abdullah *et al.* [59] using the SCAPS-1D simulator, who explored the use of a few kesterite-based materials, that is CZTS, Cu_2_ZnSnSe_4_ (CZTSe) and Cu_2_NiSnS_4_ (CNTS), where CZTSe performed the best, with an efficiency of 12.91%.

The outstanding optoelectronic properties of cadmium telluride (CdTe), including a direct bandgap of approximately 1.45 eV at room temperature, have made it a widely used material in thin-film SCs. This bandgap closely matches the solar spectrum, enabling efficient conversion of sunlight into electrical power. In addition, CdTe exhibits a high absorption coefficient, allowing it to absorb over 90% of incident sunlight within a thin layer (approx. 1–2 µm). Its bandgap is also close to the optimal value predicted by the Shockley–Queisser limit for single-junction SCs, contributing to its commercial success in thin-film PVs [60,61]. Although tellurium (Te) is scarce and cadmium (Cd) poses carcinogenic risks, making CdTe relatively expensive and hazardous, increasing recycling efforts have significantly reduced production costs [62,63]. Recycling CdTe from end-of-life modules enables the recovery of valuable semiconductor materials and mitigates environmental risk, while its repurposing in next-generation SC technologies supports a circular economy and reduces material toxicity and reliance on newly extracted raw materials, despite large-scale recycling infrastructure remaining largely at the pilot-project stage [64,65]. To the best of our knowledge, despite its advantages and extensive use in various SC technologies, CdTe has not yet been explored as a solid-state hole transport material in DSSCs.

This study investigates the performance of CdTe and three kesterite materials, that is copper-iron-tin sulfide (CFTS), CBTS and copper-manganese-tin sulfide (CMTS), as HTLs in various ssDSSC configurations using SCAPS-1D software. The device architecture incorporated FTO as the TCO, ZnO as the electron transport layer (ETL), N719 as the sensitizing dye and platinum (Pt) as the back contact. The thickness of the HTLs and the dye has been optimized within a specific range. Employing SCAPS-1D software offers a strategic approach to reduce experimental complexity by enabling virtual optimization of device parameters. SCAPS makes it easier to identify the best device topologies and appropriate material combinations by simulating a variety of structural and compositional variants.

## 2. Design and modelling of the proposed solid-state dye-sensitized solar cell configurations

### 2.1. Numerical analysis employing SCAPS-1D simulator

The computational model makes it easier to understand how SCs work and what factors are most important for improving their efficiency. Using the SCAPS-1D software, important one-dimensional semiconductor equations can be solved through numerical methods [66–69]. One of these, Poisson's equation [70], describes how the electric potential is related to the charge distribution in the material, as shown in equation (2.1).

(2.1) $$\frac{d^{2}}{dx^{2}}\psi =\frac{q}{{ɛ}_{0}{ɛ}_{r}}\;\left[p\left(x\right)-n\left(x\right)N_{D}-N_{A}\rho_{p}-\rho_{n}\right].$$

In this context, the electrostatic potential is represented by ψ, with $\varepsilon_{r}$ and $\varepsilon_{0}$ denoting the relative permittivity of the material and the permittivity of free space, respectively. *N_D_* and *N_A_* correspond to the densities of ionized donors and acceptors, while *n* and *p* indicate the electron and hole concentrations. The spatial charge distributions of electrons and holes are denoted by $\rho_{n}$ and $\rho_{p}$, and *q* represents the elementary charge. The continuity equation, which is acknowledged as a fundamental governing equation, incorporates the combined effects of drift, diffusion, recombination and carrier production. For precise modelling of carrier transport in semiconductors, a comprehensive representation is essential. Equations (2.2) and (2.3) offer a numerical elucidation of the fluctuations in electron and hole density over time.

(2.2)∂n∂t=1q∂Jn∂x(Gn−Rn),(2.3)∂p∂t=1q ∂Jp∂x(Gp−Rp).

In this context, $J_{n}$ and $J_{p}$ represent the current densities of electrons and holes, respectively. Similarly, *G_n_* and *G_p_* indicate their generation rates, while *R_n_* and *R_p_* denote the recombination rates. The drift-diffusion model, detailed in equations (2.4) and (2.5), outlines carrier transport by combining electric field-driven drift and diffusion driven by concentration gradients. These equations are essential for modelling and simulating the dynamics of charge carriers in PV devices.

(2.4)Jn=qμnnεqDn∂n,(2.5)Jp=qμppεqDp∂p,

where carrier mobilities are indicated by *µ*_*n*_ and *µ*_*p*_ for electrons and holes, respectively, while the corresponding diffusion coefficients are represented by *D_n_* and *D_p_*. According to the Einstein relation, the diffusion coefficient is intrinsically linked to both the carrier mobility and lifetime [71]. Furthermore, the absorption coefficient for the films was evaluated using the recently refined √*E_g_* model, which is an updated adaptation of the conventional √ (hυ −  *E_g_*) formulation. This relationship is formally described by the ‘Tauc laws’, as presented in equation (2.6) [72,73].

(2.6) $$\alpha \;\left(h\upsilon \right)=\left(\alpha_{0}\frac{\beta_{0}\;E_{g}}{h\upsilon }\;\right)\;.\;\sqrt{\frac{h\upsilon }{E_{g}}-1}.$$

In this formulation, hυ represents the photon energy, while  *E_g_* denotes the material's bandgap, and α corresponds to the absorption coefficient. Equations (2.7) and (2.8) establish the relationship between the revised model parameters α_0_ and β_0_ and the traditional constants *A* and *B*, thereby relating the modified absorption model to its conventional counterpart as demonstrated below.

(2.7)α0= A  √Eg,(2.8) β0=B Eg.

### 2.2. Device structure

The structure of a ssDSSC is composed of several layers, as shown in figure 1a. The cell starts with a TCO layer as the front contact, made from FTO, which allows light to enter the cell. A thin layer of zinc oxide (ZnO) nanoparticles is deposited on the TCO, providing a large surface area for dye attachment and facilitating electron transport. The dye is responsible for absorbing sunlight. Ruthenium complex dye (N719) is widely used for this purpose [47]. When the dye releases an electron to the FTO semiconductor layer, it becomes oxidized. The p-type solid-state HTL then donates an electron to the dye, restoring it to its original state so it can absorb light again [51,74]. Finally, platinum (Pt) has been used as a counter electrode that completes the circuit. It facilitates electron flow back from the external circuit to the HTL, enabling the recombination of electrons with the holes. During the preliminary analysis, approximately 8–10 conventional and kesterite (Cu_2_XSnS_4_) compounds were screened as HTLs for ssDSSCs. Most were excluded owing to unfavourable band alignment with the dye/ZnO interface, availability of material data and numerical instability in SCAPS-1D simulations. CdTe, CFTS, CBTS and CMTS exhibited stable convergence and suitable band alignment; consequently, four device architectures employing these four compounds as the HTL are investigated in this study. Therefore, the layer arrangement of these experimental models is FTO/ZnO/N719/HTL/Pt, as illustrated in figure 1a. The energy bandgap of each layer is illustrated in figure 1b.

**Figure 1.**
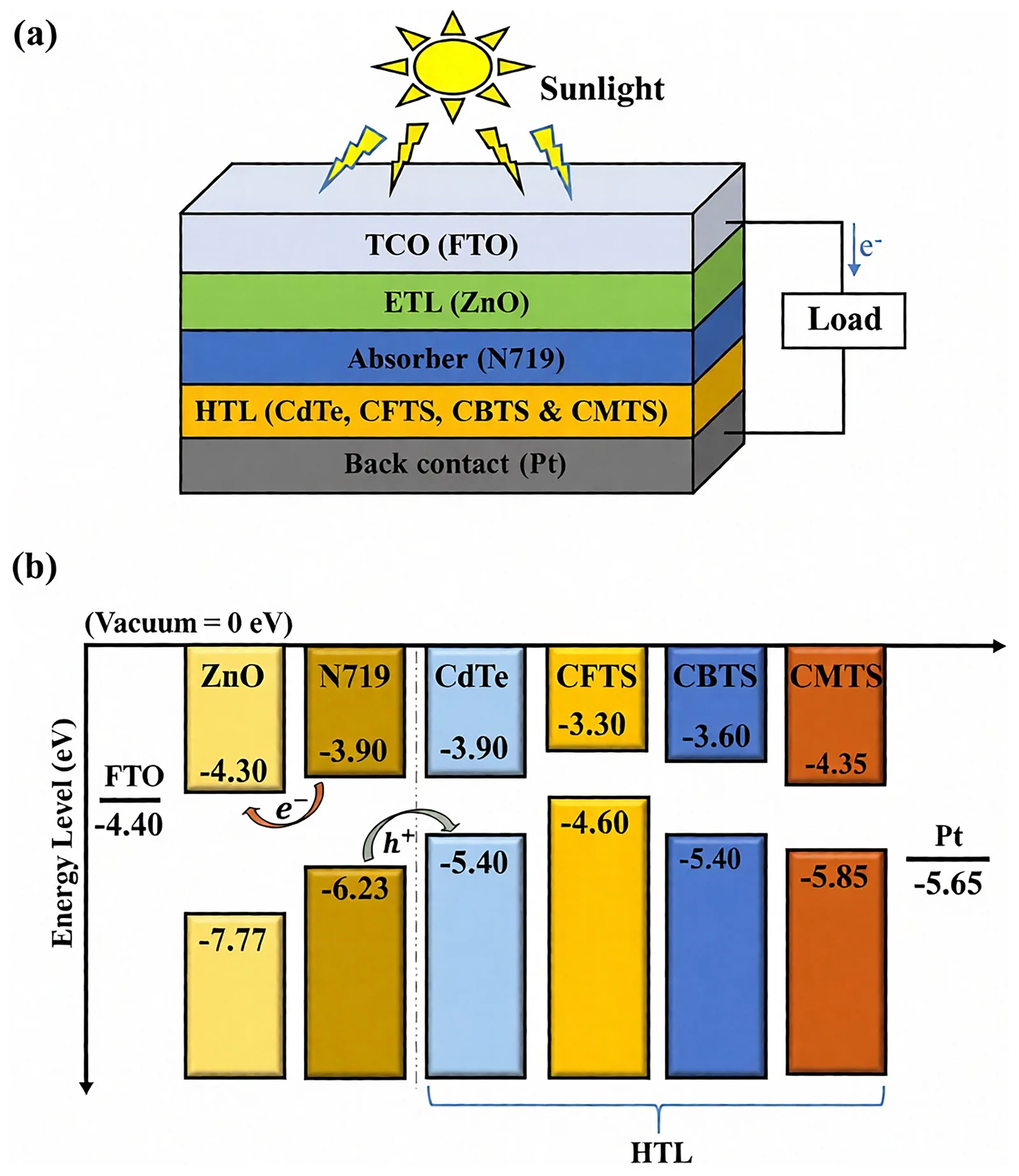
(a) Crystal structure and (b) energy band alignment between different layers.

### 2.3. Simulation using SCAPS-1D software

The input parameters configured in the simulation for the FTO layer, absorber layer, ETLs and HTL are shown in table 1. The thickness of the N719 dye absorber and the HTLs were varied relative to each other while keeping the defect density (*N_t_*) and acceptor concentration (*N_A_*) constant. Four distinct SC structures were developed based on ZnO as the ETL and N719 dye as the absorber, each incorporating a different HTL. The thickness was adjusted for each structure to identify the optimal value under these conditions. The interfacial defect parameters at both the ZnO/N719 and N719/HTL interfaces were kept at their default values as provided by the simulation software. The defects were assumed to be electrically neutral, with identical electron and hole capture cross-sections of 1.0 × 10⁻¹⁹ cm². A single-level energetic distribution was considered, with energy levels positioned above the valence band (VB) maximum. The interface defect density was uniformly set at 1.0 × 10¹⁰ cm⁻² for both interfaces. These default parameters were retained to facilitate accurate modelling of charge carrier dynamics. To determine the best performance, all output parameters were analysed. Following this, additional parameters, such as temperature and bandgap, were varied to assess their influence on the behaviour of the designed structures. The optimal operating temperature of the SC is experimentally determined by altering the temperature, as SCs generally perform most efficiently near room temperature, a finding of significant scientific importance. All simulations are conducted under standard conditions, with the ambient temperature fixed at 300K, an operating frequency of 1 MHz and illumination based on the AM1.5G solar spectrum.

**Table 1.** Input optimization parameters of different layers employed in this study.

				HTLs			
properties	FTO [53,59]	ZnO [51,75]	N719 [59,76]	CdTe [77,78]	CFTS [72]	CBTS [72]	CMTS [79]
thickness (nm)	300	50	40–200	300	300	300	300
bandgap, *E_g_* (eV)	3.60	3.47	2.33	1.50	1.30	1.90	1.50
electron affinity, *X* (eV)	4.00	4.30	3.90	3.90	3.30	3.60	4.35
dielectric permeability (relative), * Ɛ_r_*	9.00	9.00	30	9.40	9.00	5.40	9.00
conduction band effective density of states, *N_C_* (cm^−^^3^)	2.2 × 10^18^	3.7 × 10^18^	2.4 × 10^20^	8.0 × 10^17^	2.2 × 10^18^	2.2 × 10^18^	2.2 × 10^18^
valence band effective density of states, *N_v_* (cm^−^^3^)	1.8 × 10^19^	1.8 × 10^19^	2.5 × 10^20^	1.8 × 10^19^	1.8 × 10^19^	1.8 × 10^19^	1.8 × 10^19^
electron thermal velocity (cm s^−1^)	1.0 × 10^7^	1.0 × 10^7^	1.0 × 10^7^	1.0 × 10^7^	1.0 × 10^7^	1.0 × 10^7^	1.0 × 10^7^
hole thermal velocity (cm s^−1^)	1.0 × 10^7^	1.0 × 10^7^	1.0 × 10^7^	1.0 × 10^7^	1.0 × 10^7^	1.0 × 10^7^	1.0 × 10^7^
electron mobility, *µ_n_* (cm^2^ V^−1^ s^−1^)	20	100	5.00	320	21.98	30	16
hole mobility, *µ_h_* (cm^2^ V^−1^ s^−1^)	10	25	5.00	40	21.98	10	16
shallow uniform acceptor density, *N_A_* (cm^−3^)	0.00	0.00	1.0 × 10^17^	1.0 × 10^14^	1.0 × 10^18^	1.0 × 10^18^	1.0 × 10^17^
shallow uniform donor density, *N_D_* (cm^−3^)	1.0 × 10^19^	1.0 × 10^18^	0.00	0.00	0.00	0.00	0.00
defect density, *N_t_* (1/cm^3^)	1.0 × 10^15^	1.0 × 10^15^	1.0 × 10^15^	1.0 × 10^15^	1.0 × 10^15^	1.0 × 10^15^	1.0 × 10^15^

## 3. Result and analysis

### 3.1. Effect of dye and hole transport layer thickness on cell performance

The performance of DSSCs is strongly influenced by the thickness of both the dye layer and the HTL. An optimal dye thickness is crucial for maximizing light absorption, which enhances the short-circuit current density (*J*_SC_); however, excessively thick dye layers can lead to increased charge recombination and hinder electron transport. Similarly, the HTL thickness plays a vital role in efficient hole extraction and transport. A thin HTL may lead to inadequate surface coverage and increased charge recombination, whereas an excessively thick HTL can result in higher series resistance, which adversely affects the fill factor (FF) and overall power conversion efficiency. In this study, the thickness of the HTL was varied from 100 to 300 nm and the absorber thickness from 40 to 200 nm, and the open-circuit voltage (*V*_OC_), short-circuit current density (*J*_SC_), FF and efficiency of the four previously mentioned ssDSSC configurations were calculated. The results are illustrated in the contour plots presented in figures 2–5. *V*_OC_ was maximum for each device at an HTL thickness of 300 nm and at a varied absorber thickness from 40 to 200 nm. Among all devices, *V*_OC_ was found to be a maximum of 1.52 V at a 40 nm thickness of the N719 absorber and a 100 nm thick CBTS HTL, as depicted in figure 2c. CdTe and CMTS showed a slightly smaller *V*_OC_ of just above 1.171 V at an absorber and HTL thickness of above 140 and 100 nm, as shown in figure 2a,d, respectively. CFTS showed its highest *V*_OC_ of 0.67 V at an absorber thickness of 120 nm and an HTL thickness of 260 nm or above, as depicted in figure 2b, which is the minimum among the four HTL materials. Since the built-in potential formed by the four chosen HTLs is not equal, as defined by their electron affinity value, which is between 3.30 and 4.35 eV, the *V*_OC_ of each device also shows quite different values. This difference in terms of *V*_OC_ between devices employing different HTL materials is mainly owing to their bandgap difference and also because of their higher series resistance at different thicknesses. However, the *V*_OC_ of all the devices tends to increase with the increase in absorber thickness but shows a mixed trend in the case of HTL thickness, as seen in figure 2. This is because these materials have a higher series resistance and a lower photocurrent at various HTL thicknesses. In certain cases, an increased thickness of the HTL layer may obstruct efficient charge extraction and facilitate recombination, which consequently leads to a decrease in *V*_OC_, as shown in figure 2a.

**Figure 2.**
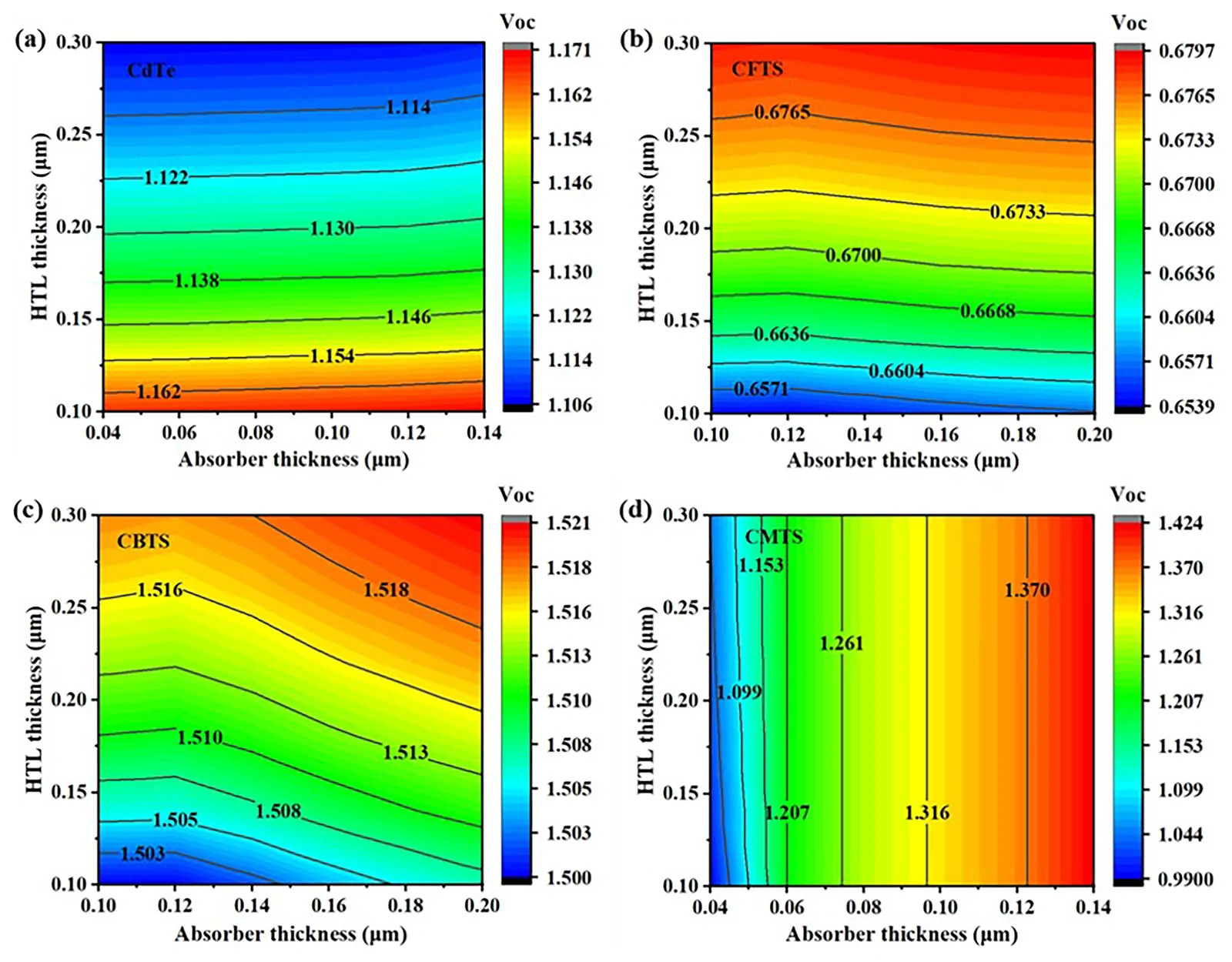
Contour mapping of *V*_OC_ for different thicknesses of N719 dye absorber and HTLs ([a] CdTe, [b] CFTS, [c] CBTS and [d] CMTS).

**Figure 3.**
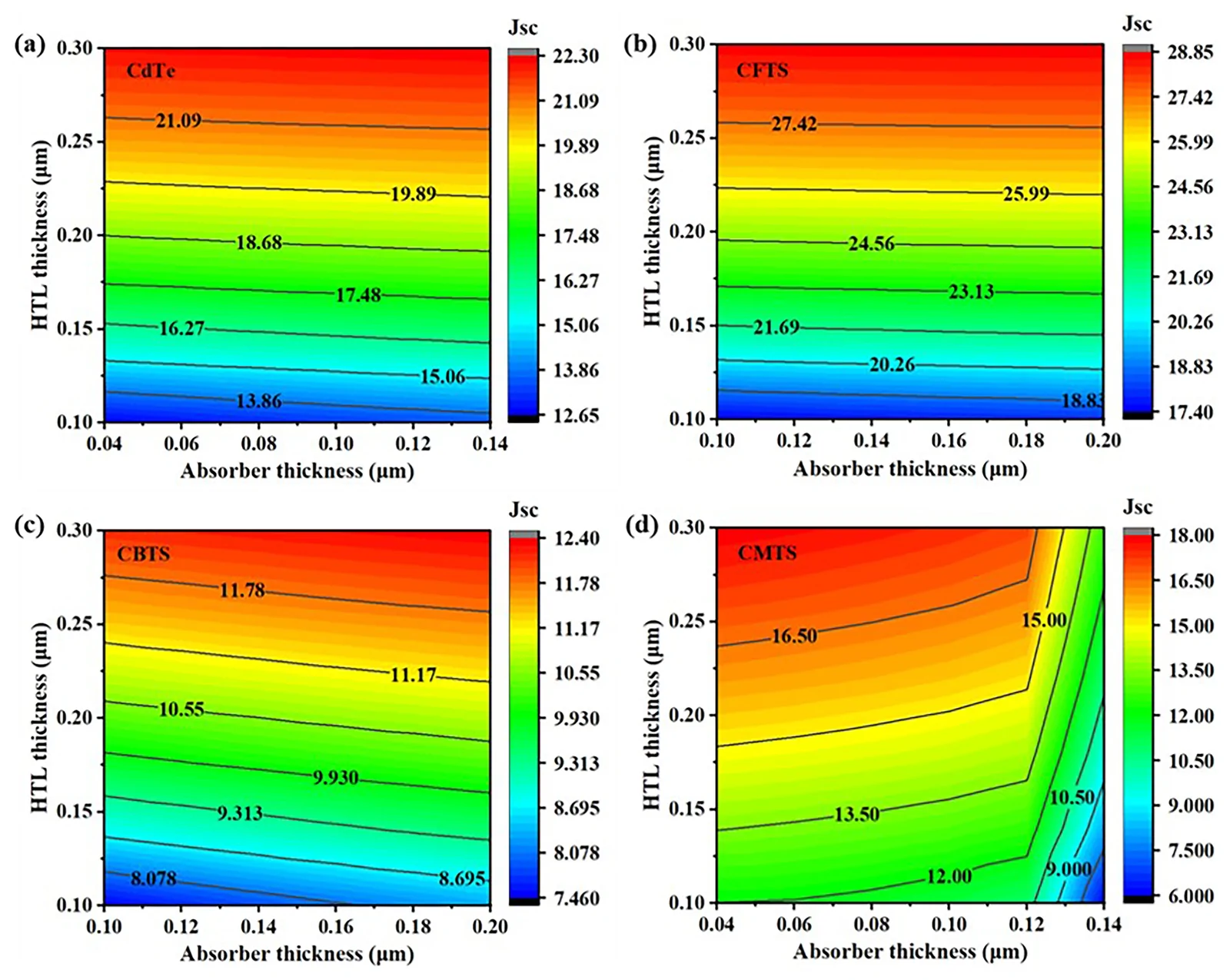
Contour mapping of *J*_SC_ for different thicknesses of N719 dye absorber and HTLs ([a] CdTe, [b] CFTS, [c] CBTS and [d] CMTS).

**Figure 4.**
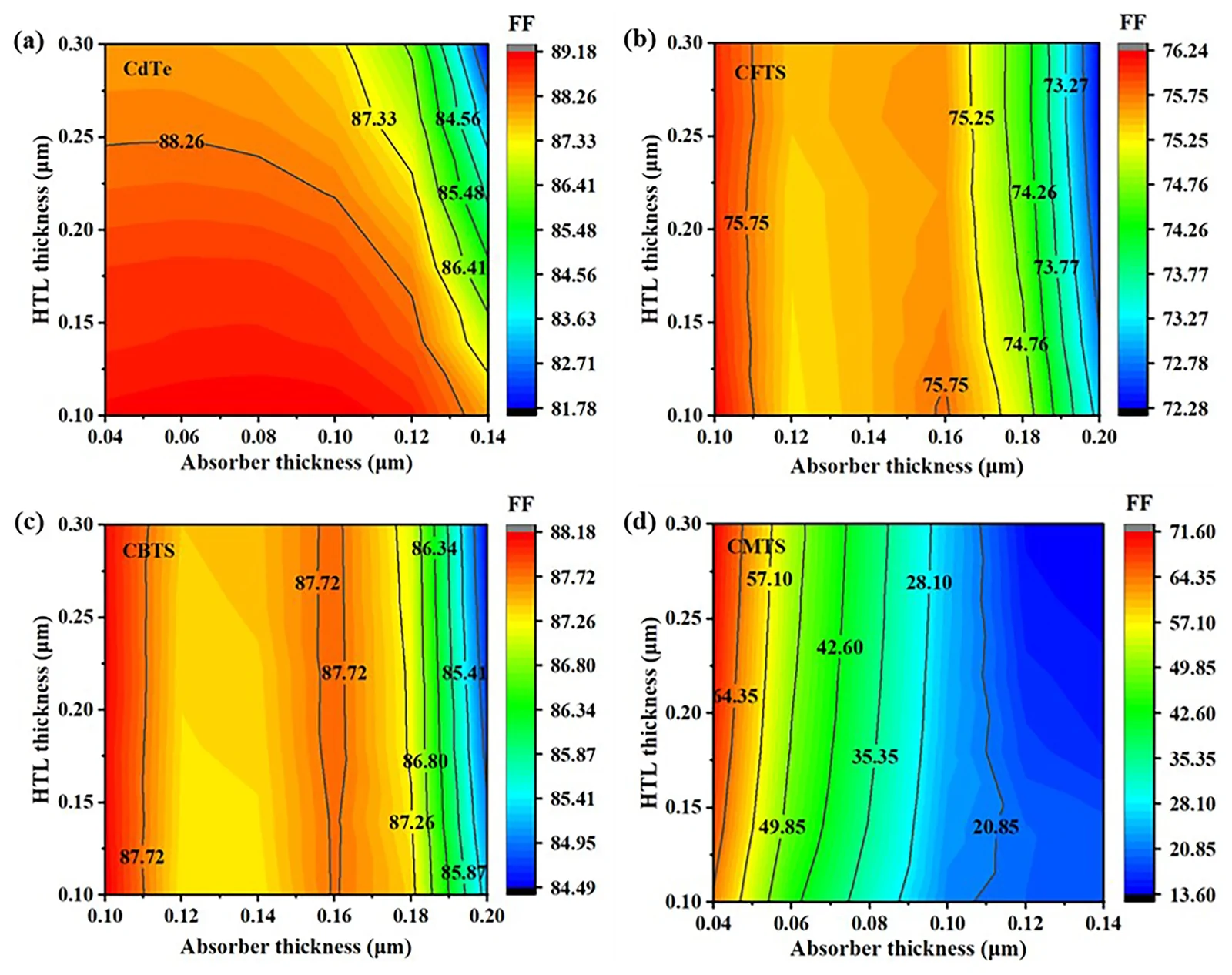
Contour mapping of FF for different thicknesses of N719 dye absorber and HTLs ([a] CdTe, [b] CFTS, [c] CBTS and [d] CMTS).

**Figure 5.**
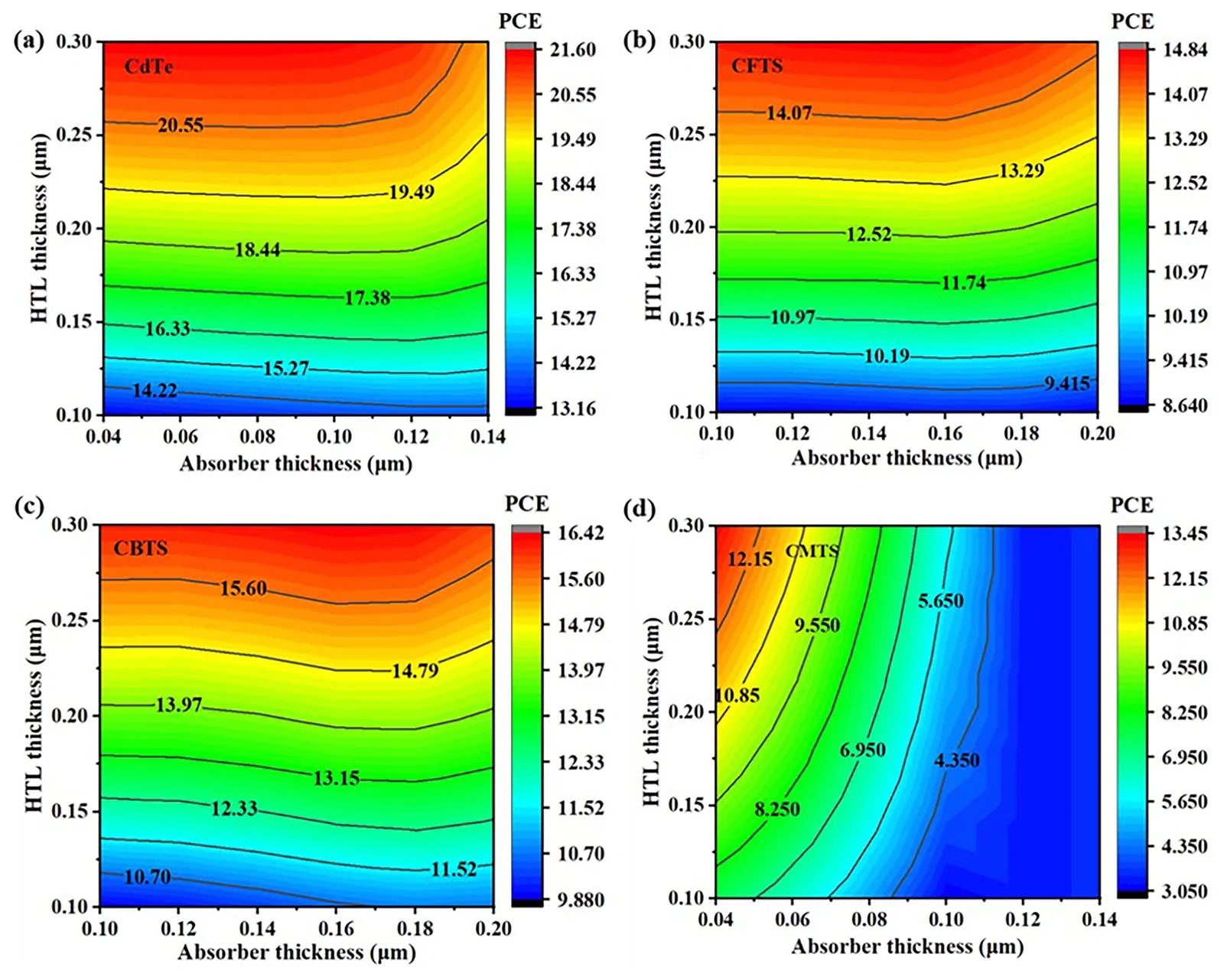
Contour mapping of PCE for different thicknesses of N719 dye absorber and HTLs ([a] CdTe, [b] CFTS, [c] CBTS and [d] CMTS).

The *J*_SC_ for different HTL configurations is illustrated in figure 3. The highest *J*_SC_ was observed in the range of approximately 12.40–28.85 mA cm^−2^ at an HTL thickness of approximately 250 nm and a dye thickness of 140–200 nm, except for the CMTS. The highest *J*_SC_ of 28.85 mA cm^−2^ was obtained by CFTS HTL with a thickness of greater than or equal to 260 and 200 nm of absorber thickness among all four studied ssDSSC structures, as seen in figure 3b. On the contrary, a smaller *J*_SC_ of 12.40 mA cm^−2^ was obtained by CBTS HTL with an absorber thickness of 200 nm and HTL thickness of just above 250 nm, as depicted in figure 3c. The relatively higher *J*_SC_ observed in CFTS- and CdTe-based structures is owing to their higher conduction and valence band compared with the dye, resulting in the allowance of the higher number of holes to be recombined in the absorber dye layer and an improved *J*_SC_. However, CdTe, CFTS and CBTS show a similar trend: *J*_SC_ decreases owing to the increase in the HTL thickness and increases owing to the increase in the dye thickness. The *J*_SC_ increased with absorber thickness owing to enhanced spectral sensitivity at longer wavelengths, except for the structure with CMTS, which shows the exact opposite trend, and it might be owing to its band alignment and very high electron affinity compared with the others. Typically, *J*_SC_ decreases with increasing HTL thickness owing to partial light absorption. However, in some cases, a moderately thicker HTL can capture longer-wavelength photons more effectively, which can enhance *J*_SC_ by facilitating better charge extraction and minimizing recombination.

Figure 4 illustrates the FF for different HTL configurations, where the highest FF was observed in the range of approximately 89.18–71.60%, corresponding to the absorber and HTL thickness of different levels. CdTe obtained the highest FF of 89.18% when HTL thickness is minimum and the dye thickness is approximately 80 nm, as shown in figure 4a. CBTS and CFTS obtained 88.18 and 76.24% FF, respectively, as depicted in figure 4c,b. They both showed a common nature, that is, at an absorber thickness ranged between 100 and 120 nm and HTL thickness at 100 and 300 nm, they obtained their highest FF and then it falls when absorber thickness increases, and at absorber thickness of around 160 nm with minimum HTL thickness, both reach their highest FF value again. After that, it decreases again. In the case of CBTS, it shows its highest FF at 40 nm of absorber thickness and greater than or equal to 150 nm HTL thickness, that is 71.60%, and it is the lowest among all four HTLs, as shown in figure 4d. Increasing the thickness of the HTL layer decreases the FF value, indicating a potential rise in charge recombination or internal resistance within the device, which adversely affects FF.

The percentage PCE of the designed ssDSSC structures is depicted in figure 5. Among the four structures analysed, the CdTe HTL demonstrated the highest efficiency, specifically at an absorber thickness of 80–90 nm and an HTL thickness of approximately 280–300 nm, as illustrated in figure 5a. Among the three kesterite HTL materials, CFTS and CBTS obtained their highest efficiency of approximately 14.82 and 16.42% at an absorber thickness of 155–165 nm and an HTL thickness above 260 nm, as depicted in figure 5b,c, respectively. CBTS obtained the lowest efficiency among all four HTLs, that is 13.45% when the absorber thickness is below 40 nm and the HTL thickness is above 250 nm, as shown in figure 5d. Overall, when absorber and HTL thickness increased, the PV performance increased up to a certain point, since the PCE is a function of the *J*_SC_, *V*_OC_ and FF parameters.

### 3.2. Current density–voltage and quantum efficiency characteristics

The current density–voltage (J–V) and quantum efficiency (QE) characteristics of the ssDSSC structure (FTO/ZnO/N719/HTLs/Pt) with four different HTLs are illustrated in figure 6. A variable DC voltage (ranging from 0 to 1.7 V) was applied to analyse the J–V characteristic. Among the configurations, the (FTO/ZnO/N719/CFTS/Pt) structure produced the highest photocurrent, peaking around 0.69 V, while the (FTO/ZnO/N719/CBTS/Pt) structure showed the lowest at 1.51 V, as illustrated in figure 6a. The current density for the CFTS HTL reached 28.5 mA cm^−^² at 0 V and gradually dropped to zero just after passing 5 V. For the CdTe HTL, the current density remained steady at 22.5 mA cm^−^² from 0 to 1 V, then began to decline, reaching zero at 1.1 V. In the case of CBTS HTL, the current density remained stable at 12.5 mA cm^−^² until 1.4 V, before decreasing to zero at 1.51 V. These variations in the J–V curves reflect the differences in bandgaps and electron affinities of the four materials, influencing their electrical performance.

**Figure 6.**
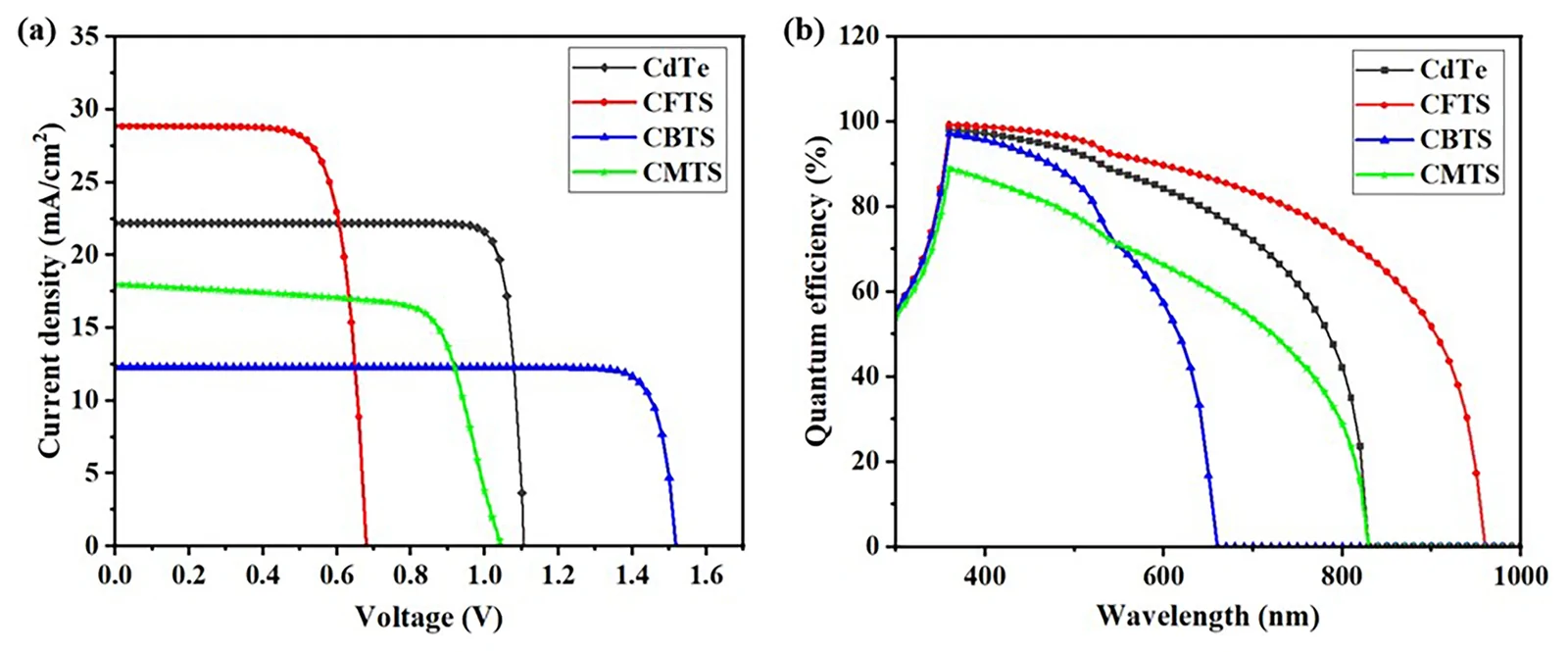
Effect of (a) J–V and (b) QE for designed ssDSSC configurations.

Figure 6b shows the relationship between wavelength and QE for the four proposed ssDSSC configurations with CdTe, CFTS, CBTS and CMTS as HTLs. QE, which depends on the light's wavelength (λ), represents the ratio of charge carriers generated to the incident photons. The wavelength was varied from 300 to 1000 nm to compare the performance of these materials. The CFTS-based configuration demonstrated the highest QE, nearly 97% at 380 nm, gradually decreasing to zero at 980 nm. CdTe and CBTS showed peak QE at the same wavelength but dropped to zero at shorter wavelengths (830 and 640 nm, respectively). CMTS exhibited lower QE overall and declined to zero even earlier. Though an ideal QE curve is depicted as square-shaped, these ssDSSC structures exhibit distinct wavelength-dependent deviations because of variations in the absorption profile of the dye, charge injection efficiency and recombination losses during carrier transport and collection. As a result, the QE spectrum reflects wavelength-dependent carrier collection probabilities rather than an ideal square response. In conclusion, CFTS offers a broader wavelength range for charge carrier generation owing to its thicker absorber layer, while CdTe and CBTS showed similar yet more limited QE ranges [80].

### 3.3. Effect of temperature variation, series resistance and shunt resistance of different hole transport layers on cell performance

#### 3.3.1. Effect of series resistance

Series resistance in SCs arises from three primary factors: first, the current flow through the interfaces between the ETL and dye, as well as between the dye and HTL; second, the resistance at the interface between the metal contact and the FTO layer; and third, the resistance of the front and rear metal contacts. Series resistance primarily impacts the FF, reducing it, and in cases of extremely high resistance, it can also negatively affect the short-circuit current (*J*_SC_), thereby diminishing overall SC performance. The open-circuit voltage (*V*_OC_) remains essentially unaffected by series resistance, owing to the absence of current flow under open-circuit conditions, while the short-circuit current (*J*_SC_) exhibits only minor sensitivity to variations in series resistance as the current–voltage curve near short-circuit is relatively flat.

Figure 7 illustrates the impact of series resistance (*R_S_*) on the performance of the ssDSSC, with a constant shunt resistance (*R*_Sh_) of 10^5^ Ω cm² to approximate a near-ideal shunt condition and minimize the influence of leakage current pathways on device performance, over an *R_S_* range of 0–6 Ω cm². Among the four key performance parameters of solar cells, that is open-circuit voltage (*V*_OC_), short-circuit current (*J*_SC_) and FF, the FF was most significantly affected by the changes in *R_S_*. As *R_S_* increased from 0 to 6 Ω cm², the FF showed a substantial decline, dropping from 87.5 to 72%, 87.5 to 82.5%, 76 to 55% and 64% for the ssDSSC configuration with CdTe, CBTS, CFTS and CMTS HTLs, while *V*_OC_ and *J*_SC_ remained relatively unchanged across all devices. The FTO/ZnO/N719/CFTS/Pt and FTO/ZnO/N719/CMTS/Pt structures exhibited comparatively poor performance, as their FF reduced significantly. However, the FTO/ZnO/N719/CdTe/Pt and FTO/ZnO/N719/CBTS/Pt structures demonstrated the least change in FF, respectively. Overall, the increase in *R_S_* has a pronounced impact on FF, and FF negatively affects the conversion efficiency in ssDSSC with different configurations.

**Figure 7.**
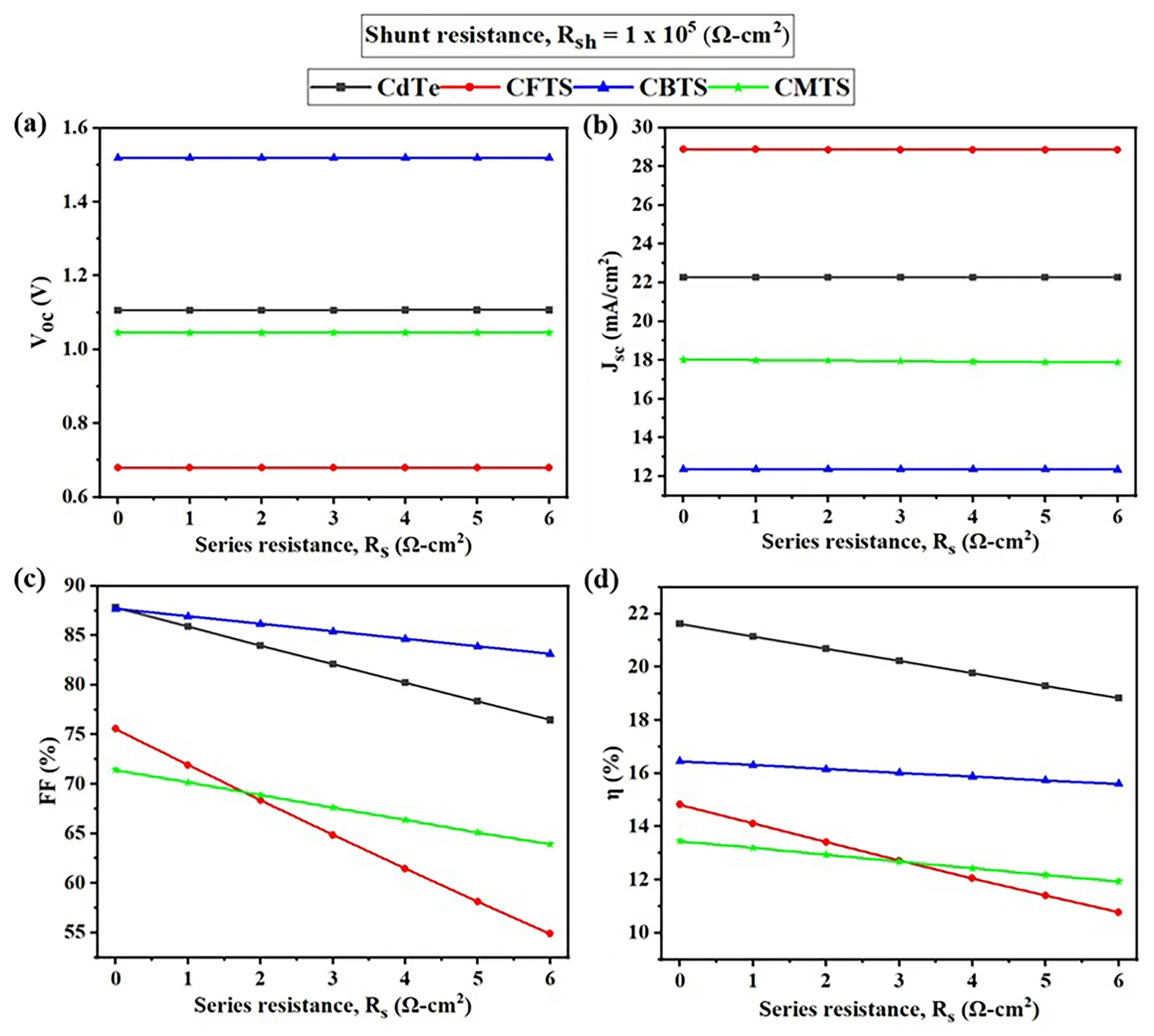
Effect of series resistance, *R_S_*, on (a) *V*_OC_, (b) *J*_SC_, (c) FF and (d) *η*%.

#### 3.3.2. Effect of shunt resistance

Power losses in SCs owing to shunt resistance are mostly driven by leakage current and non-geminate recombination. Practical cells often experience partial shorts owing to flaw growth and metal infiltration at junctions. Low shunt resistance allows current to bypass the intended path, reducing both the voltage and current through the SC. This effect is particularly pronounced under low light conditions, where the light-generated current is lower, amplifying the impact of shunt resistance.

Figure 8 illustrates the impact of shunt resistance (*R*_Sh_) in the range of 10–10⁷ Ω cm² on the PV performance of the FTO/ZnO/N719/HTL/Pt heterostructure of ssDSSC, with a fixed series resistance (*R_S_*) of 0.5 Ω cm². The figure shows significant changes in key PV parameters, that is open-circuit voltage (*V*_OC_), short-circuit current density (*J*_SC_), FF and PCE, as *R_Sh_* varies. Notably, as *R*_Sh_ increases from 10 to 10^2^ Ω cm², these parameters, especially *V*_OC_, FF and PCE, see a substantial improvement, with PCE rising from below 3% to approximately 22% for CdTe HTL. The highest PCE of around 22% is observed when *R*_Sh_ reaches 10^3^ Ω cm², maintaining this level up to 10⁷ Ω cm² or higher. Therefore, a high value of *R*_Sh_ greater than 10^3^ Ω cm² is preferable for achieving the highest PCE in the examined ssDSSC devices.

**Figure 8.**
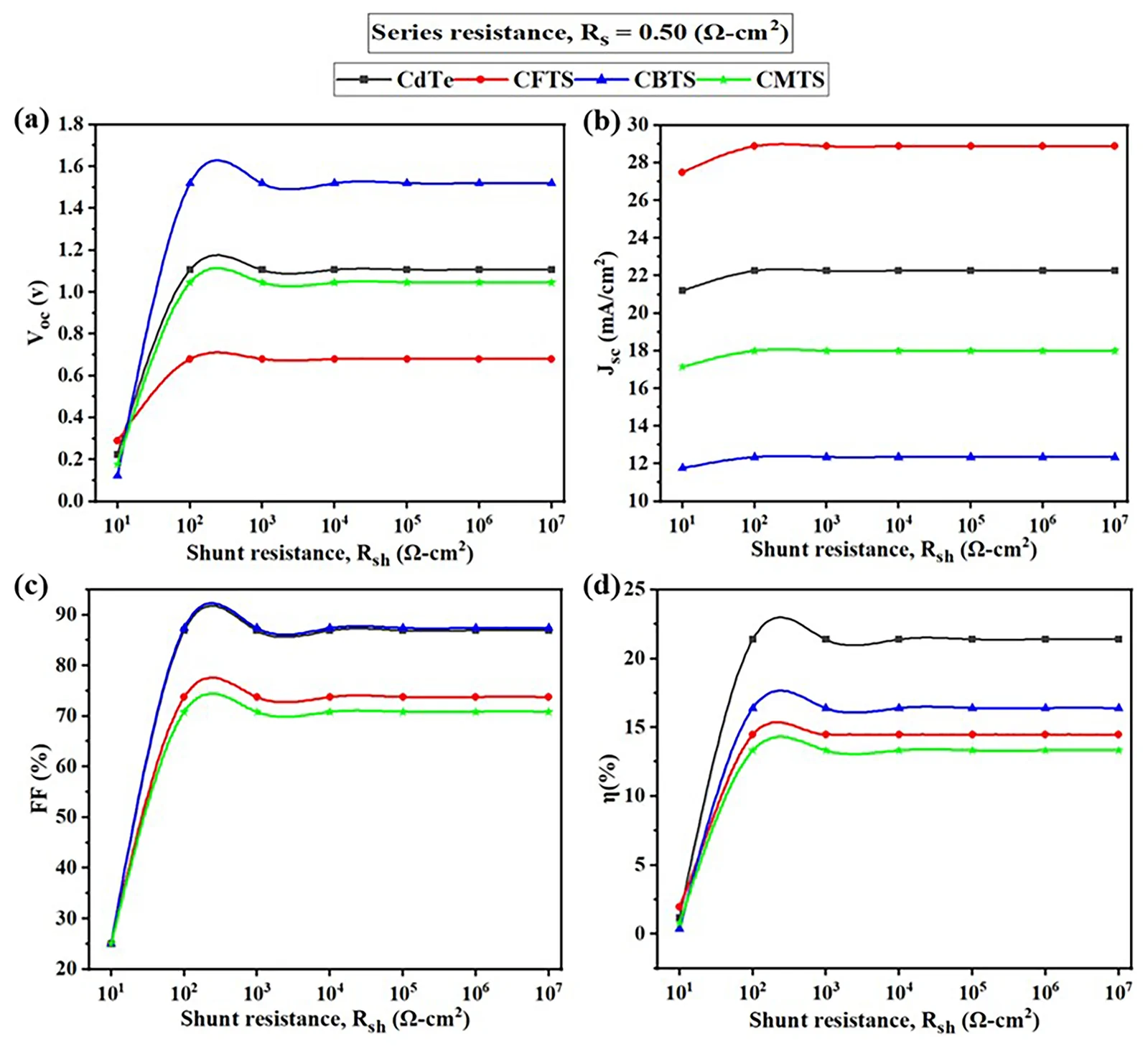
Effect of shunt resistance, *R*_Sh_, on (a) *V*_OC_, (b) *J*_SC_, (c) FF and (d) *η*%.

#### 3.3.3. Effect of temperature

The effect of temperature variations on key performance metrics, that is *V*_OC_, *J*_SC_, FF and PCE across four different ssDSSC configurations, is illustrated in figure 9. These metrics are measured at operating temperatures ranging from 275 to 475K for an FTO/ZnO/N719/HTL/Pt heterojunction ssDSSC under 1000 W m^−^² solar illumination. In conventional SCs, the parameter most significantly impacted by rising temperatures is the open-circuit voltage (*V*_OC_), which decreases as temperature increases. This reduction is owing to a steady rise in saturation current as the temperature climbs. Additionally, as temperature increases, the intrinsic carrier concentration shifts, leading to higher recombination rates. These enhanced recombination rates further contribute to the rise in saturation current, thereby affecting overall device performance [81].

**Figure 9.**
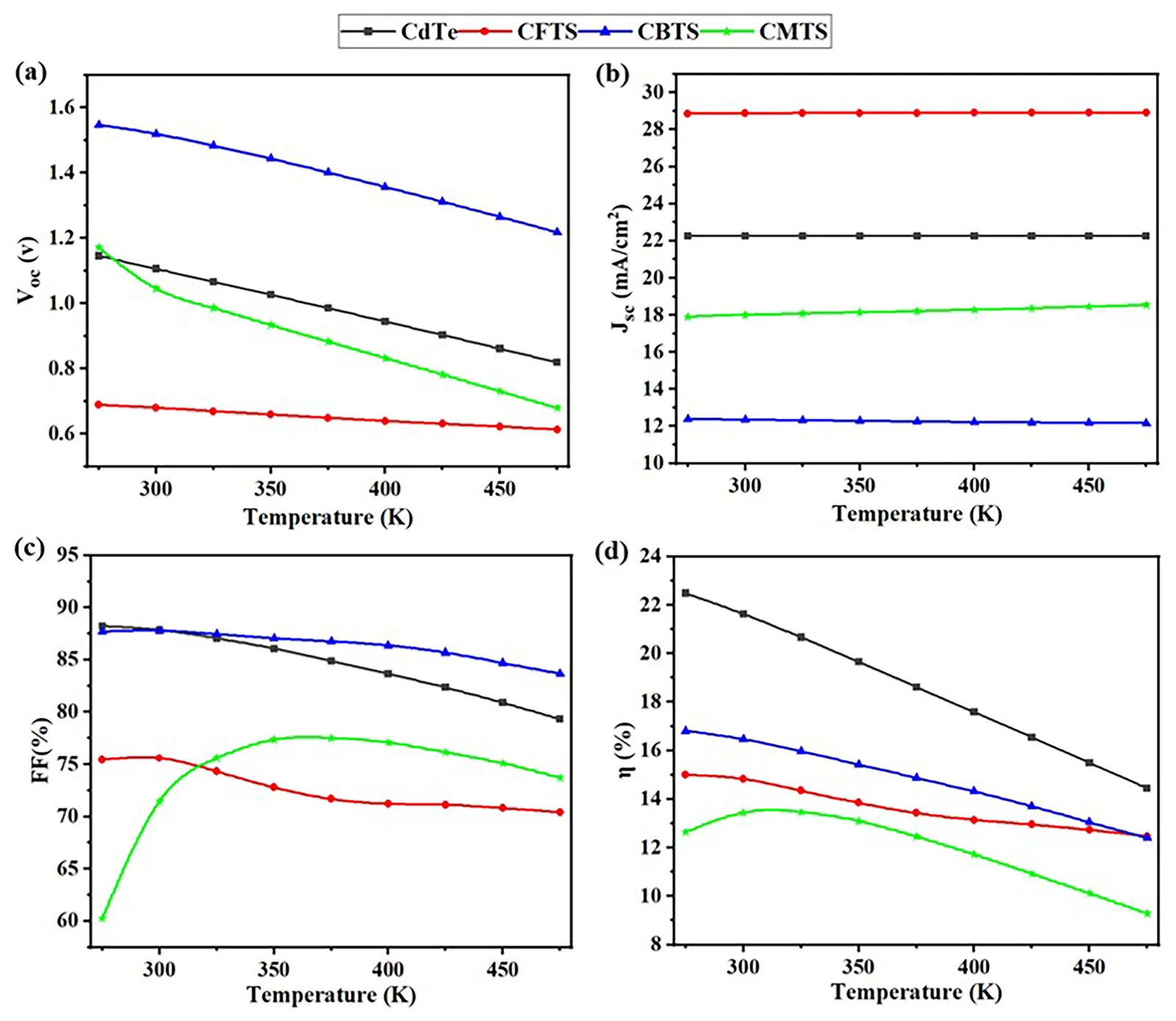
Effect of variation in temperature on (a) *V*_OC_, (b) *J*_SC_, (c) FF and (d) *η*%.

The relationship between *V*_OC_ and temperature (*T*) can be described by equation (3.1) [82],

(3.1) $$V_{\mathrm{oc}}=\frac{E_{a}}{q}-\frac{\frac{MkT}{q}\ln\left(J_{00}\right)}{J_{\mathrm{PD}}}.$$

In this context, *T* denotes temperature, *M* is the ideality factor, *E_a_* indicates the activation energy for recombination, *q* is the elementary charge, *k* is the Boltzmann constant, *J*_00_ is a prefactor, and *J*_PD_ represents the photocurrent density. Based on equation (3.1), setting the temperature *T* to 0K allows for the determination of the activation energy *E_a_*, under the assumption that *M*, *J*₀₀ and *J*_PD_ remain temperature-independent [83].

In figure 9a, it is evident that as temperature rises, the open-circuit voltage (*V*_OC_) declines, while the short-circuit current (*J*_SC_) remains virtually unchanged, showing only a negligible variation. This slight change in *J*_SC_ can effectively be disregarded. Since the FF and power conversion efficiency (*η*%) are both dependent on *V*_OC_ and *J*_SC_, a reduction in *V*_OC_ leads to a corresponding decrease in FF and PCE, as demonstrated in figure 9c,d. The primary reason for this performance decline is the increase in reverse saturation current, which escalates with higher temperatures. Among the different configurations, the CdTe-based HTL experiences a notable drop in efficiency, from 22.5 to 15%, yet it continues to outperform the other configurations in terms of overall efficiency. Within the kesterite group of HTLs, CBTS shows superior performance compared with the others, although its efficiency decreases to 13% at 475K, matching the efficiency of CFTS at that temperature. Interestingly, the structure with CMTS HTL behaves somewhat differently; a slight rise in temperature increases its current density, as it gains enough energy to overcome the energy barrier between its conduction band and the LUMO of the N719 dye. However, its efficiency also begins to decline after reaching 350K.

### 3.4. Effects of capacitance, Mott–Schottky, generation and recombination rate

#### 3.4.1. Effect of generation and recombination rate

The carrier generation and recombination rates for ZnO- and N719-based ssDSSCs are illustrated in figure 10a,b over a range of 0.0–0.8 µm position. When a photon is absorbed, an electron is excited from the VB to the conduction band, leaving behind a hole in the VB, resulting in the formation of electron–hole pairs [84]. The data reveal that the generation rate peaks for all devices occur between 0.29 and 0.31 µm. Among the configurations of the proposed ssDSSC, using a CFTS HTL exhibits the highest generation rate at 0.3 µm, while the CBTS-based configuration shows the lowest. Both the CdTe and CMTS HTLs demonstrate nearly identical generation rates, slightly lower than the CFTS peak at the same wavelength position.

**Figure 10.**
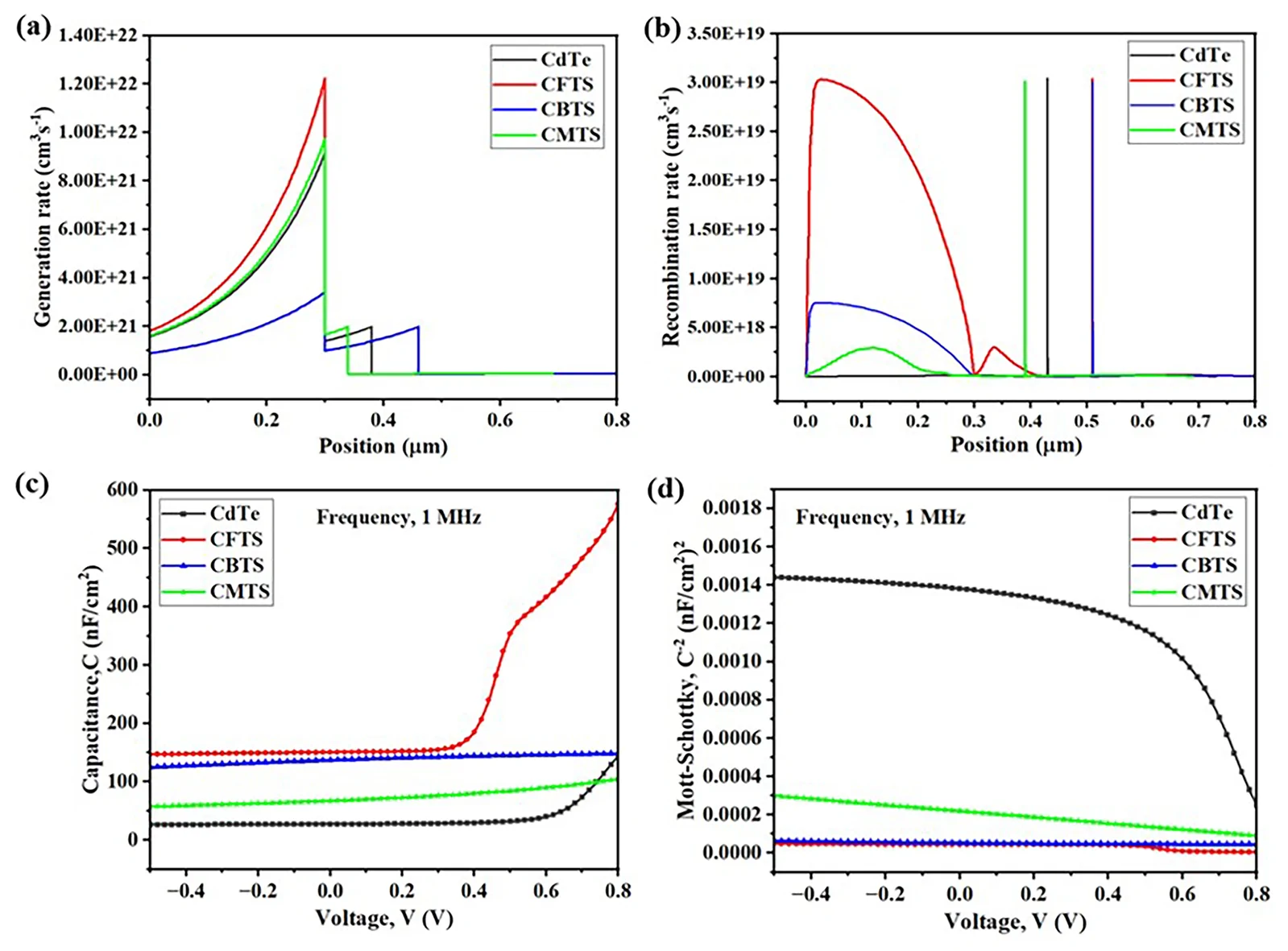
(a) Generation rate, (b) recombination rate, (c) the capacitance–voltage (*C*–*V*) response and (d) Mott–Schottky (1/*C*^2^) response of ssDSSC configurations with four HTLs.

The electron–hole pair generation rate is computed based on the incoming photon flux. This photon flux is analysed using equation (3.2), which calculates the value of the electron–hole pair generation rate for each specific wavelength and spatial location within the device, providing a detailed understanding of the carrier generation dynamics throughout the SC structure [85].

(3.2) $$G\;\left(\lambda ,x\right)=\;\alpha \left(\lambda ,x\right)\times \;N_{\mathrm{phot}}\left(\lambda ,x\right),$$

where *G* (*x*) refers to the electron–hole pair generation rate, and *N*_phot_ (*λ*, *x*) refers to the incoming photon flux.

The recombination rate, which involves the merging and annihilation of generated electrons and holes, operates in direct contrast to the carrier generation process. In ssDSSCs, the recombination rate is primarily determined by the lifetime and density of charge carriers. A reduction in electron–hole recombination is often linked to defect states within the absorber layer and at the ETL and HTL interfaces, which can trap carriers and prevent recombination. Across all the examined devices, the highest recombination rates were observed between 0.39 and 0.51 µm, with devices incorporating the CdTe and CFTS HTLs showing the most pronounced recombination peaks. The other two structures also exhibit a recombination rate close to theirs. Devices with the three kesterite candidates show another thick peak at a position just after 0 µm and slowly fall to 0 at 0.3 µm. Among them, the peak of CFTS HTL was almost the same as that of the stiff one at 0.52 µm. Electron–hole recombination is significantly influenced by mid-gap energy states that are positioned between the valence and conduction bands, leading to more noticeable recombination within the devices. Additionally, the recombination rate in ssDSSCs can exhibit non-uniform distribution, which is often attributed to grain boundaries and imperfections arising during the fabrication process. These variations in the material structure can result in localized areas of higher recombination, affecting the overall device performance.

#### 3.4.2. Effects of capacitance and Mott–Schottky

The impact of capacitance and Mott–Schottky (MS) behaviour across a biasing voltage range of −0.5 V to 0.8 V at a fixed frequency of 1 MHz for four different configurations of the proposed ssDSSC structure, each using a distinct ETL, is illustrated in figure 10c,d. In figure 10c, the capacitance of the CFTS and CdTe HTLs shows a pronounced increase when the applied voltage exceeds 0.39 and 0.6 V, respectively. Meanwhile, the other two HTLs exhibit a more gradual, linear rise in capacitance as the voltage increases. The capacitance of all the configurations maintains a constant MS relationship as the forward bias voltage is increased. Comparing the behaviour of CFTS with the other materials highlights its unique dielectric properties, suggesting distinct charge storage mechanisms in this configuration. CdTe, while similar to CFTS in its overall behaviour, requires a higher bias voltage to trigger the same capacitance changes. This indicates that both CFTS and CdTe demonstrate distinctive charge storage and dielectric characteristics, albeit at different voltage thresholds, potentially impacting the charge dynamics and performance of the ssDSSC system.

The built-in potential (*V*_bi_), which reflects the operational behaviour of electrodes and the doping level within a device, can be determined using the widely adopted MS method [86]. In this approach, the *V*_bi_ is typically identified by the *x*-axis intercept of the capacitance–voltage (*C*–*V*) curve. Additionally, the intercept of the 1/*C*²–*V* curve provides insights into the concentration of occupied trapping centres. As the *V*_bi_ increases, the corresponding MS values decrease for a given device. In this study, the curves of the capacitance–voltage (*C*–*V*) plot exhibit an increasing trend, while the corresponding MS plot shows a decreasing trend for the simulated results, which validates the accuracy of the approach.

### 3.5. Band alignment of the proposed solid-state dye-sensitized solar cell configurations with different hole transport layer

With four distinct HTLs, figure 11a–d illustrates the band alignment of different ssDSSC structures. The quasi-Fermi levels of electrons and holes are represented as *F_n_* and *F_p_*, respectively, with the conduction band minima and VB maxima of *E_C_* and *E_V_* of each layer used in the structure. Along the *x*-axis the thickness of each layer in the ssDSSC structure and along the *y*-axis the level in electron volts (eV) are given.

**Figure 11.**
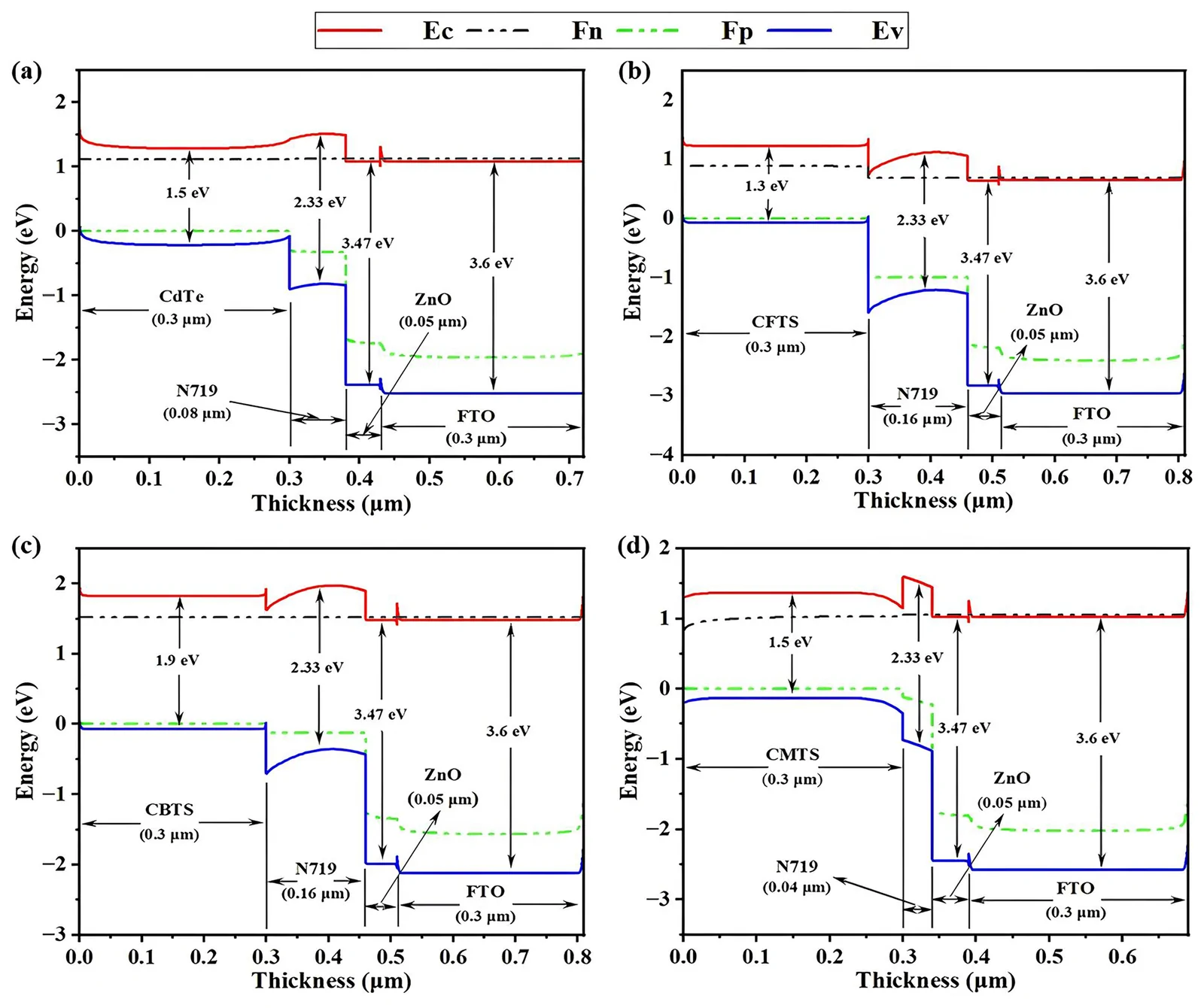
Energy band diagram of the proposed ssDSSC structure with four different HTLs: (a) CdTe, (b) CFTS, (c) CBTS and (d) CMTS.

The first three layers were kept the same in all the structures, following the cell configuration: FTO/ZnO/N719/HTLs. As a result, variations were introduced only in the HTLs, which serve as critical components in ssDSSCs by facilitating the extraction of photo-generated holes. The HOMO of the dye absorber should be distant from the ZnO conduction band, while the LUMO should be close and higher in energy. The HOMO of the dye should also be lower than that of solid-state HTL [87]. The energy levels that electrons and holes typically inhabit under illumination are represented by the quasi-Fermi levels (*F_n_* and *F_p_*), respectively. For charge carrier movement to be facilitated, *F_n_* and *F_p_* should ideally coexist between the energy bands of adjacent layers.

In all four configurations, the quasi-Fermi levels for electrons (*F_n_*) are nearly identical, located just below or equal to the conduction band minima (*E_C_*) for each layer in every combination. Similarly, the quasi-Fermi levels for holes (*F_p_*) are positioned above the VB maxima (*E_V_*) for all layers across the different structures. The conduction band level of CdTe, across all four HTLs, aligns closely with the LUMO of the N719 dye, which enhances hole extraction compared with the other materials. This suggests that CdTe is likely to demonstrate superior performance as a hole transport material.

Among kesterites, CBTS and CFTS, possessing conduction band levels higher than the LUMO of the dye, are also viable options for the proposed structure, as charge can easily pass through. Notably, the conduction band level of CBTS is closest to the dye's LUMO, suggesting its expected superior performance over CFTS. In contrast, the conduction band of CMTS is significantly lower than the LUMO of the dye, creating a substantial energy barrier that hinders charge transfer unless external energy, such as elevated temperatures, is applied. Consequently, CMTS is not an ideal hole transport material for this ssDSSC configuration, and its performance under standard conditions would be very poor, though it might show slight improvement at higher temperatures [22,25,26].

In summary, among the proposed HTLs, CdTe is expected to deliver the best performance. Among the three kesterite-based materials, CBTS is projected to perform better than CFTS and CMTS. This conclusion is further supported by previous analyses, confirming CdTe and CBTS as the most promising candidates for the proposed ssDSSC structure.

### 3.6. Output comparison with previous work

The primary objective of our research was to explore alternative solid-state HTL materials for ssDSSC in the FTO/ZnO/N719/HTL/Pt configuration. Out of the four initial candidates studied, three performed exceptionally well when compared with existing alternatives of the same category.

Table 2 provides a detailed comparison between our final simulated results and recent theoretical and simulation studies on N719-based ssDSSCs. Our simulation achieved a maximum efficiency of 21.58% with CdTe as the HTL in this configuration, which exceeds the values reported in current research. More specifically, among the kesterite candidates for the HTL, the configurations using CBTS and CFTS demonstrated efficiencies of 16.41 and 14.80%, respectively, both of which are higher than those reported by Abdullah *et al.* [59], except for CMTS.

**Table 2.** Output comparison with previous works in SCAPS-1D.

structure	*V*_OC_ (V)	*J*_SC_ (mA cm^−2^)	FF (%)	PCE (%)	year
FTO/ZnO/N719 dye/CdTe/Pt	1.106	22.21	87.86	21.58	this work
FTO/ZnO/N719 dye/CFTS/Pt	0.679	28.82	75.58	14.80	this work
FTO/ZnO/N719 dye/CBTS/Pt	1.519	12.30	87.78	16.41	this work
ITO/PC_61_BM/N719 dye/PEDOT:PSS/Pt	1.069	22.38	86.64	20.73	2024 [47]
FTO/ZnO/N719 dye/X60/Au	1.545	7.318	84.83	9.60	2024 [51]
FTO/ZnOS/N719 dye/CZTSe/Au	0.875	20.83	70.86	12.91	2024 [59]
FTO/ZnOS/N719 dye/CFTS/Au	0.555	16.84	59.09	5.53	2024 [59]
FTO/PFN:Br/ZnPC:PC70BM/PEDOT:PSS/Au	0.858	27.45	62.77	14.61	2023 [52]
FTO/PC_61_BM/N719 dye/CuSCN/Au	0.885	8.563	70.94	5.38	2021 [53]
FTO/TiO_2_/N719 dye/CuI/Au	1.452	26.50	48.12	17.72	2019 [76]

Therefore, this study not only highlights promising materials for ssDSSC configurations but also sets the stage for future exploration of impactful materials in this domain.

## 4. Conclusion

This study systematically evaluated cadmium tellurite (CdTe) and three kesterite compounds (CFTS, CBTS and CMTS) as solid-state HTLs in DSSCs (ssDSSCs) using the SCAPS-1D simulator. Device performance was examined under varying structural and operating conditions, including HTL and dye thickness, parasitic resistances and temperature effects. Among the investigated HTLs, CdTe achieved the highest power conversion efficiency (21.58%) at optimized thicknesses, attributed to favourable band alignment with the N719 dye LUMO level and efficient carrier transport. CBTS and CFTS also showed promising efficiencies (16.41% and 14.80%, respectively), whereas CMTS exhibited inferior performance owing to unfavourable band positioning and limited charge transport.

The results confirm that device performance is strongly governed by interfacial energetics and defect tolerance, identifying CdTe as the most efficient HTL candidate. Nonetheless, CBTS represents a compelling alternative owing to its cost-effectiveness, Earth-abundant composition and thermal stability. Overall, these findings demonstrate that CdTe and kesterite-based HTLs, particularly CBTS, hold strong potential for advancing ssDSSC technologies. Future work should focus on experimental validation and material optimization to translate these simulation insights into scalable, sustainable PV devices.

## Supplementary Material



## Data Availability

All device simulations in this work were carried out using SCAPS-1D software (v. 3.3.10), developed at the University of Gent, Belgium. All simulation data generated in this study have been deposited in the Zenodo repository [88], ensuring that any interested researcher can reproduce or reinvestigate the work. Supplementary material is available online [89].
